# Intraoperative radiation therapy induces immune response activity after pancreatic surgery

**DOI:** 10.1186/s12885-021-08807-3

**Published:** 2021-10-12

**Authors:** Yun Sun Lee, Hyung Sun Kim, Yeona Cho, Ik Jae Lee, Hyo Jung Kim, Da Eun Lee, Hyeon Woong Kang, Joon Seong Park

**Affiliations:** 1 Department of Surgery, Gangnam Severance Hospital, Yonsei University College of Medicine, 20, Eonju-ro 63 gil, Gangnam-gu, Seoul, 06229 South Korea; 2 Brain Korea 21 FOUR Project for Medical Science, Yonsei University, Seoul, South Korea; 3 Department of Radiation Oncology, Yonsei University College of Medicine, Seoul, South Korea

**Keywords:** Pancreatic cancer, Intraoperative radiation therapy, Immune response, Cytokine

## Abstract

**Background:**

Pancreatic cancer has highly aggressive features, such as local recurrence that leads to significantly high morbidity and mortality and recurrence after successful tumour resection. Intraoperative radiation therapy (IORT), which delivers targeted radiation to a tumour bed, is known to reduce local recurrence by directly killing tumour cells and modifying the tumour microenvironment.

**Methods:**

Among 30 patients diagnosed with pancreatic cancer, 17 patients received IORT immediately after surgical resection. We investigated changes in the immune response induced by IORT by analysing the peritoneal fluid (PF) and blood of patients with and without IORT treatment after pancreatic cancer surgery. Further, we treated three pancreatic cell lines with PF to observe proliferation and activity changes.

**Results:**

Levels of cytokines involved in the PI3K/SMAD pathway were increased in the PF of IORT-treated patients. Moreover, IORT-treated PF inhibited the growth, migration, and invasiveness of pancreatic cancer cells. Changes in lymphocyte populations in the blood of IORT-treated patients indicated an increased immune response.

**Conclusions:**

Based on the characterisation and quantification of immune cells in the blood and cytokine levels in the PF, we conclude that IORT induced an anti-tumour effect by activating the immune response, which may prevent pancreatic cancer recurrence.

**Clinical trial registration:**

NCT03273374.

**Supplementary Information:**

The online version contains supplementary material available at 10.1186/s12885-021-08807-3.

## Background

Pancreatic cancer is reportedly one of the most aggressive cancers and the fourth leading cause of cancer-related deaths [1]. Despite improvements in treatment, the 5-year survival of patients remains < 5%. Moreover, although curative resection is the only choice for pancreatic cancer, it has been insufficient in improving long-term survival [2]. Alternatively, radiation therapy could provide a treatment option to increase the survival rate of patients with pancreatic cancer by inducing the immune activity of cytokines.

The conventional external beam radiation therapy (EBRT) of pancreatic cancer is extremely challenging. Its effectiveness is limited because adequate radiation cannot be provided owing to the low tolerance of adjacent organs [3, 4]. Recent advanced radiotherapy techniques, such as intensity-modulated radiotherapy (IMRT), image-guided radiotherapy (IGRT), magnetic resonance (MR)-guided RT, and particle therapy have improved outcomes in pancreatic cancer patients [5–7]. However, these treatments are limited to patients with unresectable or borderline resectable pancreatic cancer, and their clinical use as adjuvant to conventional treatments has not yet been established. Intraoperative radiation therapy (IORT) using a portable instrument delivers a single fraction of high-dose radiation during surgery [8, 9] that effectively focuses intense radiation to a desired site and simultaneously reduces toxicity to normal tissues [4, 10].

A high dose of radiation triggers immunogenic cell death and stimulates anti-cancer immune response [11, 12]. Irradiation activates IFN-dependent immunity via the formation of DAMP molecules and upregulates the expression of pro-inflammatory cytokine genes by regulating NF-kB [13]. These cytokine cascades are radiation dose-dependent and have shared characteristics. A number of studies have described the effects of various complex components in the inflammatory microenvironment that increase the therapeutic effect by controlling the dynamic anti-cancer immune response [14, 15]. Therefore, the anti-tumour immune response could be an important mechanism responsible for modulating the immuno-suppressive microenvironment.

The only prospective randomised control trial to investigate the effectiveness of IORT to date was performed in Japan, although an increased survival was not confirmed [16]. However, other studies have shown that IORT significantly improves local control and overall survival in patients with pancreatic cancer by reducing post-operative complications and mortality [17, 18]. High-dose irradiation directly induces DNA damage in irradiated cells and changes the tumour microenvironment. According to Kulcenty et al., IORT not only alters the composition of anti-tumour-related cytokines in the surgical wound fluid, but also affects the tumorigenic properties of breast cancer [19]. Thus, research studies are currently underway to decipher the effects of IORT on the tumour microenvironment, but little is known about pancreatic cancer.

The aim of this study was to investigate changes in the tumour microenvironment after IORT and its effects on pancreatic cancer. We also examined changes in pancreatic cancer cell proliferation and invasiveness using peritoneal fluid (PF) samples from IORT-treated patients with pancreatic cancer. This study improves our understanding of how IORT-induced changes in the tumour microenvironment alter the immune response and induce anti-cancer immune activity in patients with pancreatic cancer.

## Methods

### Patient selection

This single-institution prospective phase II study was approved by the institutional review board (3–2015-0102) and written informed consent was obtained from each patient. Thirty patients diagnosed with pancreatic cancer at the Gangnam Severance Hospital from 2018 to 2019 underwent pancreatic resection (Supplementary Table S1, Supplementary Table S2). The eligibility criteria were as follows: 1) age 20 years or older; 2) histologically or clinically confirmed pancreatic carcinoma; 3) Eastern Cooperative Oncology Group (ECOG) performance status scores of 0–2; 4) resectable disease defined as follows: absence of distant metastases; absence of direct involvement of the inferior vena cava or aorta; and clear fat planes around the celiac axis, hepatic artery, and superior mesenteric artery; 5) stage I–III disease as per the 7th edition of the American Joint Committee on Cancer (AJCC); 6) good bone marrow function (haemoglobin level > 10 g/dL, absolute neutrophil count > 1500/mm^3^, and platelet count > 100,000/mm^3^); and 7) adequate renal function (serum creatinine level < 1.4 mg/dL and blood urea nitrogen level < 20 mg/dL). Patients who 1) had previously received RT to the abdominal area; 2) had a tumour bed that could not be adequately covered by the IORT field as defined by the radiation oncologist; 3) had received neoadjuvant chemotherapy; 4) had synchronous distant metastasis; 5) were pregnant or nursing; or 6) had any condition rendering them unsuitable for IORT (at the discretion of the physicians) were excluded from this study.

### Treatment scheme

A total of 17 patients were treated with IORT (irradiated with a single dose of 10 Gy at a depth of 5 mm into the tumour bed) immediately after surgical resection, as previously described [20]. Patients were subjected to curative resection, either pylorus-preserving pancreatoduodenectomy (PPPD), distal pancreatectomy, or total pancreatectomy. A mobile 50-kV X-ray source (Intrabeam, Carl Zeiss, Germany) was used for IORT. The target volume included the tumour bed, the celiac and superior mesenteric arteries, the mesenteric root, and the portal vein; any areas deemed at risk by the surgeon and radiation oncologist were also included. A spherical applicator with a diameter of 3.5 cm was used. An additional shielding device was attached to the spherical applicator, leaving only the bottom surface unshielded from which the X-ray beam was delivered to the tumour bed. The target volume was irradiated with a single dose of 10 Gy, prescribed at a 5-mm depth into the tumour bed.

### Peritoneal fluid collection

Patients were divided into the group that received IORT (IORT PF, *n* = 17) and the group that did not receive IORT (no IORT, *n* = 13). PF was collected from the usual surgical drain 24 h after the surgery. Sterile filtered PF samples were centrifuged at 2100 rpm for 15 min and then stored at − 80 °C.

### Blood sample collection and isolation of peripheral blood mononuclear cells

Blood was collected three times from patients (post-operation day (POD) 1, 7, and 14) using vacutainer EDTA tubes (BD Biosciences, San Jose, CA, USA). Whole blood was diluted with Dulbecco’s phosphate-buffered saline (DPBS), gently layered over an equal volume of Ficoll-Paque solution (GE Healthcare, Logan, UT, USA), and centrifuged at 400 *g* for 30–40 min. Peripheral blood mononuclear cells (PBMCs) were collected from the second layer, and any remaining platelets were gently washed off using DPBS. Cells were resuspended in freezing medium containing 10% DMSO and 90% foetal bovine serum (FBS; Biowest, Riverside, MO, USA) and stored at − 80 °C.

### Cell culture

Mia PaCa-2, Panc1, and Aspc1 cells were obtained from American Type Culture Collection (Manassas, VA, USA). Cells were cultured in DMEM or RPMI medium (Biowest) supplemented with 10% FBS (Biowest) and 1% antibiotic–anti-mycotic reagent (Gibco, Waltham, MA, USA). Cells were incubated at 37 °C in a humidified atmosphere under 5% CO_2_.

### Cytokine array

PF samples were assayed using the Human Cytokine Antibody Array kit (Abcam, Cambridge, UK) according to the manufacturer’s instructions. Briefly, each sample was incubated for 24 h at 4 °C and then paired with biotinylated detector antibodies and horseradish peroxidase (HRP)-conjugated streptavidin. Each cytokine was analysed using chemiluminescence and levels were quantified using ImageJ software (National Institutes of Health, Bethesda, MD, USA).

### Flow cytometry

Isolated PBMCs were stained with the following fluorochrome-conjugated monoclonal antibodies: anti-human APC-CD4 (eBioscience, San Diego, CA, USA), APC-cy7 CD8 (BD Biosciences, San Jose, CA, USA), PE-cy5 CD25 (Biolegend, San Diego, CA, USA), PE-Cy7 Foxp3 (eBioscience), BV421 CD56 (BD Biosciences, San Jose, CA, USA), and APC-CD19 (Biolegend). Live cells were classified using propidium iodide (BD Biosciences, San Jose, CA, USA) staining. Lymphocytes were further subtyped by their staining properties as T cells (CD3+), Th cells (CD3 + CD4+), Tc cells (CD3 + CD8+), NK cells (CD3-CD56+), or Treg cells (CD4 + CD25 + FOXP3+). Treg cells were fixed and underwent permeabilisation using the Fix & Perm Buffer (eBioscience), and stained cells were analysed using BD FACSCanto II Cell Analyzer (BD Biosciences, San Jose, CA, USA). FlowJo software (BD Biosciences, San Jose, CA, USA) was used for compensation and data analysis.

### Proliferation assays

For the 2D proliferation assay, 1.5–3 × 10^3^ cells per well were seeded into a 96-well cell culture plate. Cells were allowed to grow in serum-free medium supplemented with 5% FBS or PF. After incubation for 4 days, reduced medium with 10% WST-1 reagent (EZ-cytox, Dogen, Korea) was placed into the wells after aspiration of the growth medium. The absorbance of each well was measured at 450 nm using a VersaMax microplate reader (Molecular Devices, San Jose, CA, USA).

For the colony formation assay, 100–500 cells/well were seeded into six-well culture plates containing reduced serum medium supplemented with 5% PF and antibiotics. The medium was replaced every 3 days until colonies formed. The rate of colony formation was different for each cell, usually taking 7–14 days. Cells were fixed in cold 100% methanol for 30 min, stained using 2.5% crystal violet, and then washed several times with phosphate-buffered saline (PBS). Colony formation was calculated manually and digitally using ImageJ software, where colony area versus total area was calculated based on staining intensity.

### Migration and invasion assays

For the invasion assay, the 8 μm pore size Transwell system (Corning, Midland, MI, USA) was coated with Matrigel (1:50, Corning) at 37 °C for 1 h. Then, 2 × 10^4^ cells were seeded on the apical side of the Transwell chamber (24-well insert) in serum-free media. DMEM with 7% PF and 1% antibiotic–anti-mycotic agent was added to the basal compartment. The cells were allowed to invade for at 37 °C 24 h. The cells that remained in the top chamber were gently scraped off using wet cotton swabs. The cells that invaded the basal side were fixed in methanol for 10 min, stained with 0.2% crystal violet, and then washed multiple times with distilled water. The invasion assays were performed in triplicate.

For the scratch wound migration assay, 2 × 10^4^ cells were seeded into a 96-well plate (Image lock, IncuCyte™; Essen Bioscience, Ann Arbor, MI, USA), and wound scratches were made using a wound maker tool (Essen Bioscience) 18 h after plating. The media in each well was supplemented with 5% PF and 1% antibiotic–anti-mycotic agent, and FBS was used as positive control. Images of the migrated cells were captured automatically every 4 h, and the relative wound density was analysed using IncuCyte™ Chemotaxis Cell Migration Software (Essen Bioscience).

### Western blotting

After the indicated treatment, cells were harvested and washed with ice-cold PBS and lysed using RIPA lysis buffer. Proteins (30 μg sample) were separated using SDS-PAGE and transferred onto nitrocellulose membranes, blocked in 5% skim milk, and incubated with the following primary antibodies (1:1000): Anti-phospho-smad2, phospho-smad3, smad2, and smad3 (Cell Signaling Technology, Danvers, MA, USA), anti-Snail+slug (Abcam), anti-vimentin (Cell Signaling Technology), N-cadherin (BD Biosciences, San Jose, CA, USA), E-cadherin (BD Biosciences, San Jose, CA, USA), and γ-tubulin (Sigma-Aldrich, St. Louis, MO, USA). The membranes were then washed thrice with Tris-buffered saline and Tween 20 (TBST), followed by incubation with HRP-conjugated secondary antibody (1:7000, Cell Signaling Technology) in TBST with 3% skim milk. Bound antibody was probed using ECL solution.(Bio-Rad, USA) Chemiluminescent signals were captured using X-ray films. All experiments were performed in triplicate.

### RNA isolation and qPCR

After the indicated treatment, cells were collected, and their RNA was isolated using TRIZOL Reagent® (Sigma-Aldrich) according to the manufacturer’s instructions. Then, 0.2 μg total isolate RNA was analysed via reverse transcriptase PCR using the One-Step RT-PCR Kit (*iNtRON* Biotechnology, Seongnam-Si, Korea). First-strand cDNA synthesis was performed with 1 μg RNA as a template using the RT-qPCR cDNA Synthesis Kit (*iNtRON* Biotechnology), according to the manufacturer’s instructions. RT-qPCR was performed using the SYBR qPCR reaction mix (Applied Biosystems, Foster City, CA, USA). The primer sequences used in this study are listed in Table 1. Relative mRNA expression level was calculated using the 2^−ΔΔCT^ method, using GAPDH as the reference gene.

**Table 1:** qPCR primer sequence

Gene		5′-3′ sequences
Vimentin	Forward	CACGAAGAGGAAATCCGGAGC
	Reverse	CAGGGCGTCATTGTTCCG
SNAIL	Forward	CAAGGAATACCTCAGCCTG
	Reverse	GGCTTCTCGCCAGTGTG
E-cadherin	Forward	TGCCCAGAAAATGAAAAAGG
	Reverse	GTGTATGTGGCAATGCGTTC
N-cadherin	Forward	GGCATACACCATGCCATCTT
	Reverse	GTGCATGAAGGACAGCCTCT
GAPDH	Forward	GTCTCCTCTGACTTCAACAGCG
	Reverse	ACCACCCTGTTGCTGTAGCCAA

### Statistical methods

Statistical analysis was performed using GraphPad Prism version 8.01 software (GraphPad Software, La Jolla, CA, USA). Unpaired *t*-test was performed for statistical analysis of cytokine array, cell proliferation, western blotting, and qPCR data. Wound healing assay and PBMC phenotyping data were analysed by two-way ANOVA. Differences were considered statistically significant at * *p* < 0.05, ** *p* ≤ 0.01.

## Results

### Peritoneal fluid from IORT-treated patients displayed cytokine composition changes

**Fig. 1:**
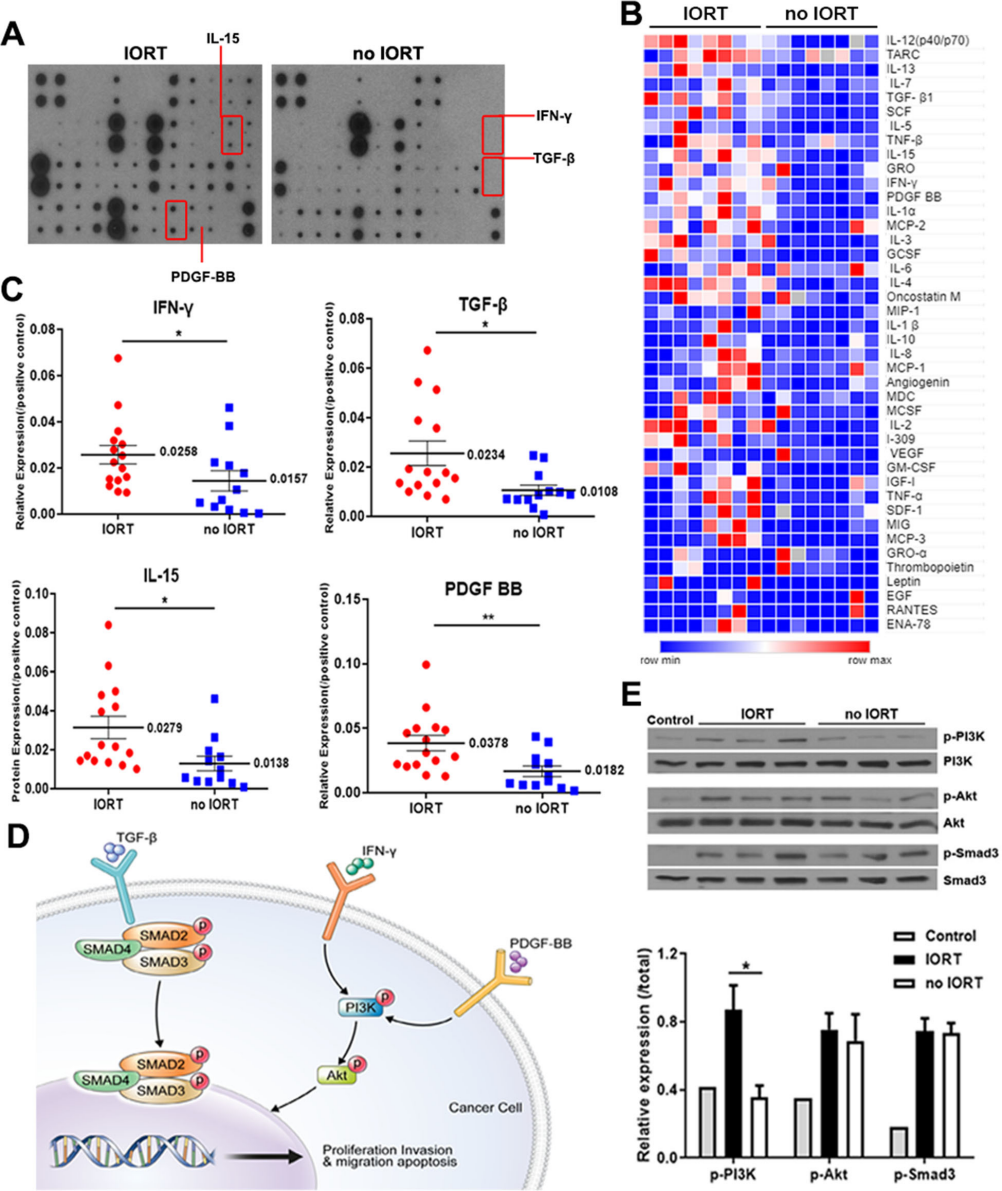
Intraoperative radiation therapy (IORT) leads to differential cytokine profile of the peritoneal fluid (PF) and affects PI3K/Smad signalling in pancreatic cancer cells. **A** Cytokine membrane array images. Each spot represents a cytokine hybridised in patient PF. **B** Heat map visualisation comparing cytokine array results. **C**. Cytokines significantly upregulated in the PF of IORT-treated patients (IORT *n* = 15, no IORT *n* = 11). **D**. Schematic overview of signalling pathways (PI3K-Akt, Smad2/3) in cancer cells associated with cytokines TGF-β, IFN-γ, and PDGF-BB. **E**. Western blot analysis of signalling-related protein xpression in Panc1 cells incubated with PF for 4 h. IORT: P3, P54, and P56; no IORT: P31, P33, and P42 (unpaired *t*-test; *p < 0.05 and **p ≤ 0.01)

We investigated cytokine changes induced by IORT in the PF of 30 patients with pancreatic cancer (Fig. 1A). Our results revealed that the relative signal intensity of 19 cytokines were higher in the IORT PF group than in the no IORT group, whereas levels of 17 cytokines were decreased (Fig. 1B, Supplementary Table S3). Among them, the levels of IFN-γ (*p* = 0.0357), IL-15 (*p* = 0.0172), platelet-derived growth factor (PDGF)-BB (*p* = 0.0042), and TGF-β (*p* = 0.0174) differed significantly with respect to the presence or absence of IORT (Fig. 1C). We further investigated whether these cytokine profile differences were related to the activation of intracellular pathways in pancreatic cancer cells (Fig. 1D). Pancreatic cancer cells incubated with IORT-treated PF for 4 h displayed activated cytokine-related signalling pathways (PI3K-Akt, Smad2/3) compared with cells incubated with no IORT-treated PF (Fig. 1E). Hence, IORT altered the cytokines secreted into the peritoneal cavity after surgery.

### IORT-treated PF suppressed pancreatic cancer cell growth

**Fig. 2:**
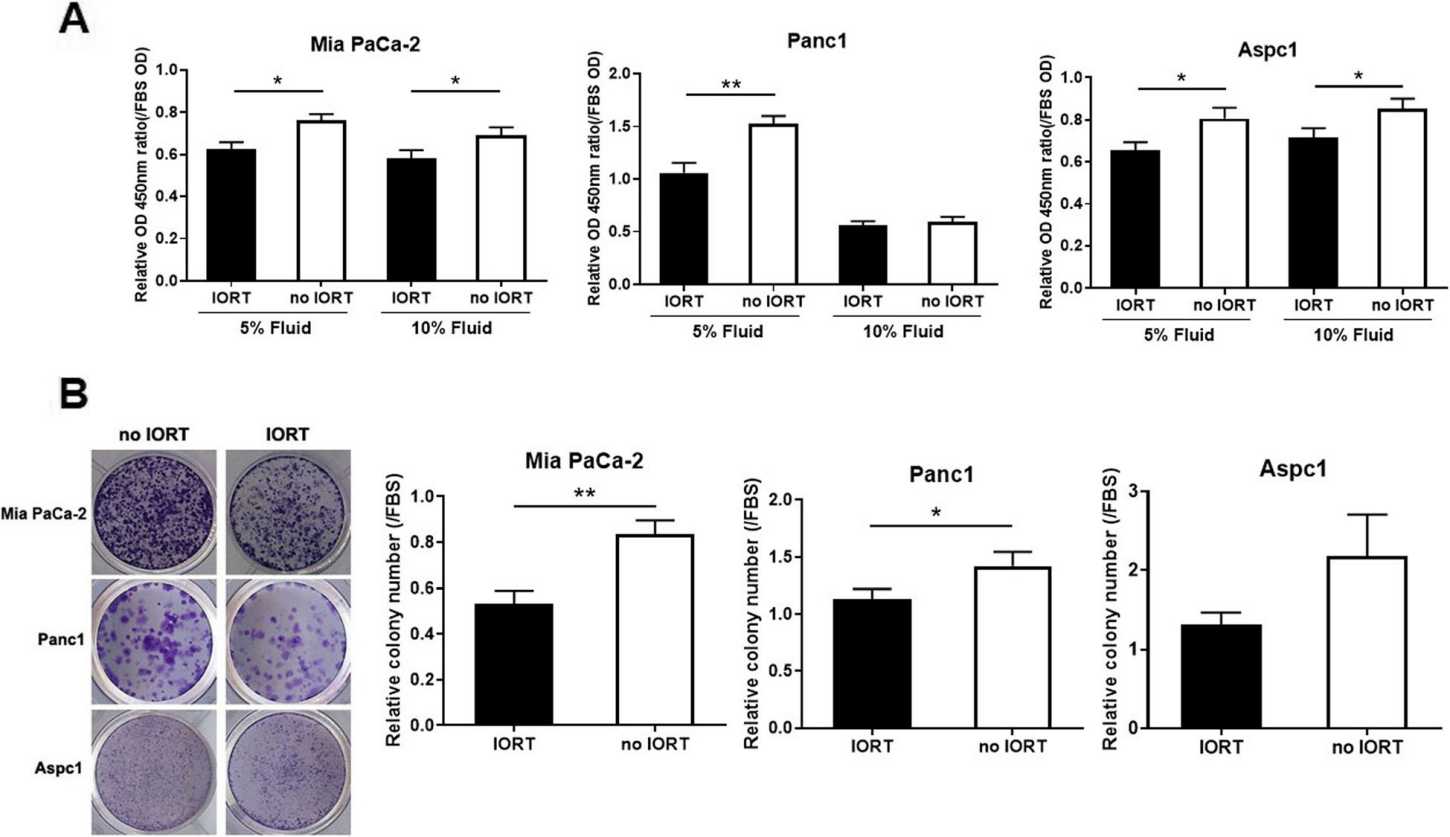
Peritoneal fluid (PF) stimulates cancer cell proliferation. **A**. Mia PaCa-2, Panc1, and Aspc1 cell lines were incubated for 4 days in the presence of PF at the indicated concentrations. As a positive control, foetal bovine serum (FBS) was used at the same concentration as PF. Incubated cells were treated with WST-1 solution, and absorbance was detected at 450 nm. **B**. Cells were incubated in the presence of PF until the colonies were formed. Colonies were stained with crystal violet, and the percentage of colony areas were calculated using ImageJ software. Columns correspond to the mean of three independent experiments; bars indicate SD. Statistical analyses were performed using the unpaired *t*-test

Next, we performed WST-1-based proliferation assays on Mia PaCa-2, Panc1, and Aspc1 cells to confirm the influence of IORT-treated PF on pancreatic cancer cell proliferation (Fig. 2A). The proliferation of pancreatic cancer cells stimulated by no IORT-treated PF was comparable to that of cells cultured in complete media (with FBS). However, pancreatic cancer cells in the IORT PF group demonstrated significantly reduced proliferation compared with those in the no IORT PF group. This difference was most pronounced in the Panc1 cells. Additionally, a similar pattern of IORT influence was displayed in the colony formation assay (Fig. 2B). PF exerted a stimulating effect on pancreatic cancer cell proliferation, which was inhibited in the IORT PF group.

### IORT-treated PF decreased invasiveness and wound healing activity of pancreatic cancer cells

**Fig. 3:**
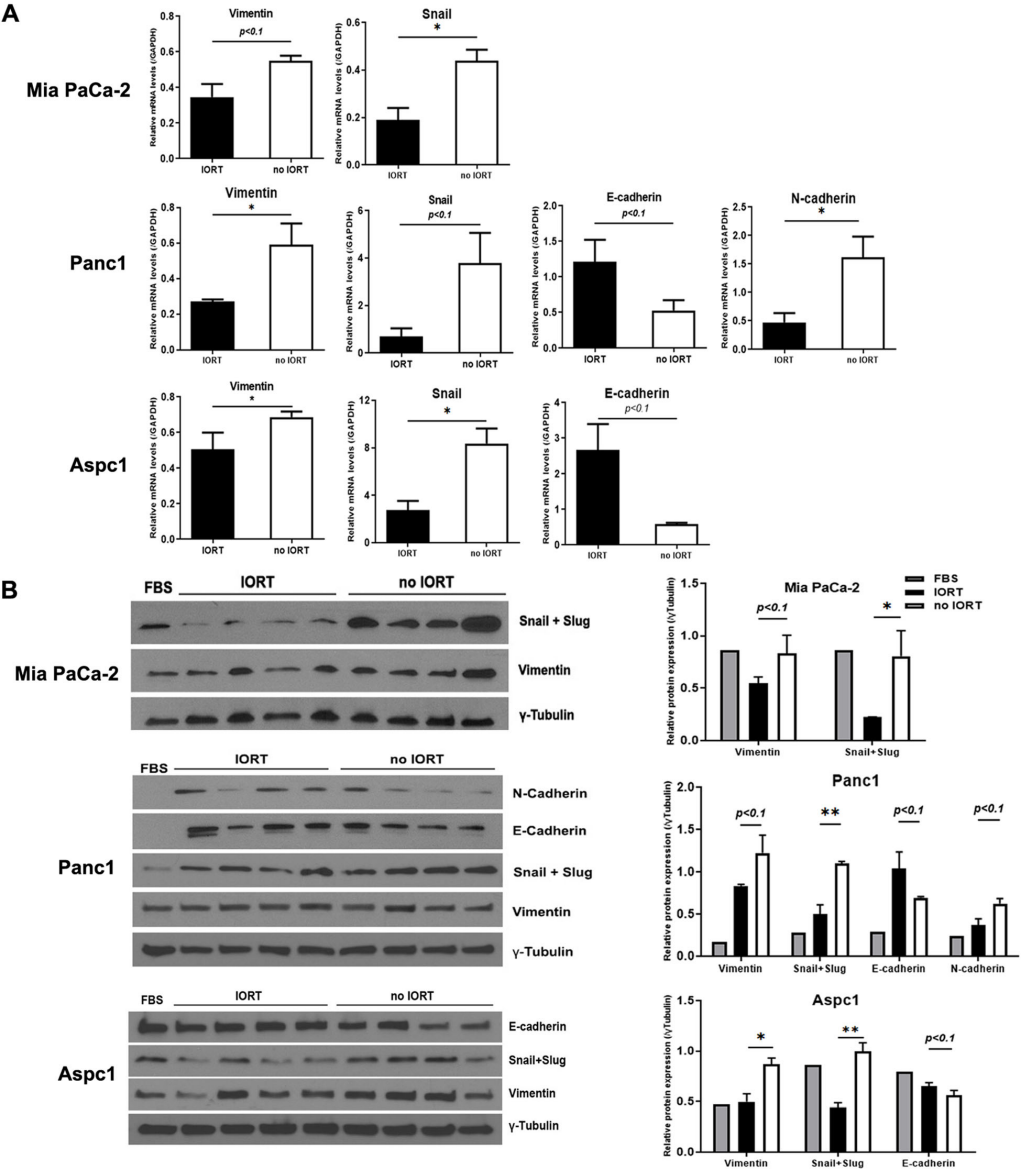
Intraoperative radiation therapy (IORT)-treated peritoneal fluid (PF) decreased the invasiveness and wound healing ability of pancreatic cancer cells. **A**. Pancreatic cancer cell lines were tested for their invasiveness using Matrigel-coated Transwell chemotaxis assays. Random PF samples (7% in DMEM) were loaded in the bottom well of the transwell plate for pancreatic cancer cell treatment. Invaded cells were stained and analysed after 24 h. **B**. Pancreatic cancer cells cultured in 96-well plates were scratched using a wound maker and treated with PF for observing cell migration every 12 h

Next, we analysed the effect of IORT-treated PF on the invasive capability of pancreatic cancer cells through a 3D Matrigel-coated Transwell-based assay using five randomly selected PF samples from each group. Incubation with IORT-treated PF reduced the invasiveness of all three pancreatic cancer cell lines (Fig. 3A). Assessing the migratory ability of cells incubated with PF over time (Fig. 3B) revealed that cells in the IORT PF group had a slower wound closure rate than those in the no IORT group.

### IORT changed the expression of epithelial-mesenchymal transition (EMT) markers in pancreatic cancer cells

**Fig. 4:**
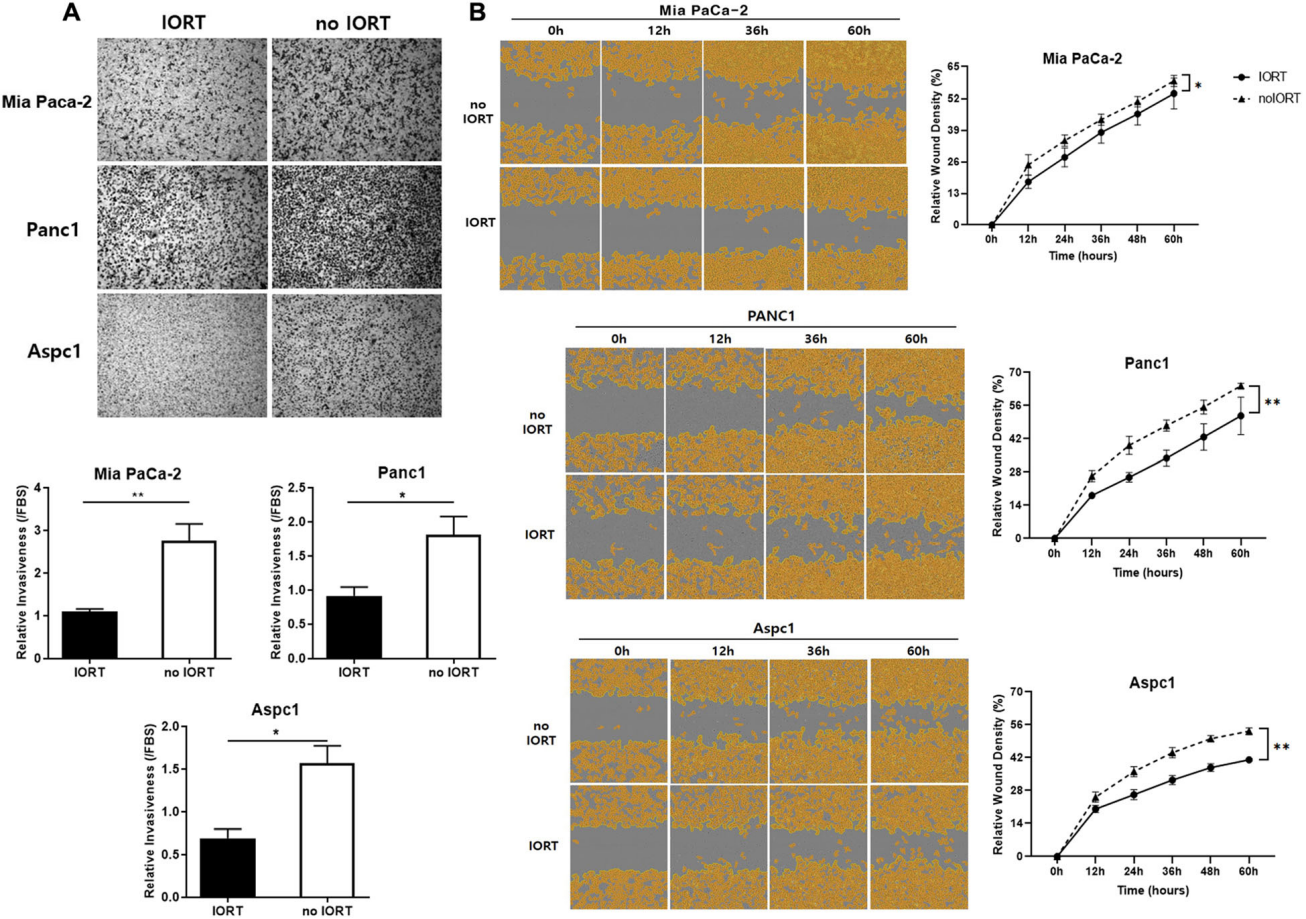
Changes in EMT marker expression in cancer cells following treatment with peritoneal fluid (PF) collected after intraoperative radiation therapy (IORT). **A**. qPCR was used to measure the expression levels of EMT-related genes encoding mesenchymal markers (vimentin, snail, and N-cadherin) and epithelial markers (E-cadherin). **B**. Western blotting analysis of the expression of indicated EMT markers. RNA and protein were extracted from cells after 4 days of treatment. Used PF were from IORT (P1, P43, P56, and P64) and no IORT (P26, P42, P45, and P62). The results were statistically analysed using the unpaired *t*-test, and significance was marked at *: *p* < 0.05 and **: *p* < 0.01 using three independent biological replicates

We treated Mia PaCa-2, Panc1, and Aspc1 cells with the same 7% PF used in the invasion assay. Comparing the average expression levels of EMT-related genes in PF-treated cells indicated that the expression of epithelial marker E-cadherin was increased, but that of mesenchymal markers vimentin, snail, and N-cadherin was decreased in the IORT PF-treated cells (Fig. 4A). According to the western blot results, expression levels of the mesenchymal markers were decreased in the IORT PF group (Fig. 4B). These data implied that treatment with IORT-treated PF regulated the expression of EMT markers in pancreatic cancer cells, ultimately inhibiting cell invasiveness and migration activity.

### Changes in lymphocyte phenotypes indicated increased immune response after IORT

**Fig. 5:**
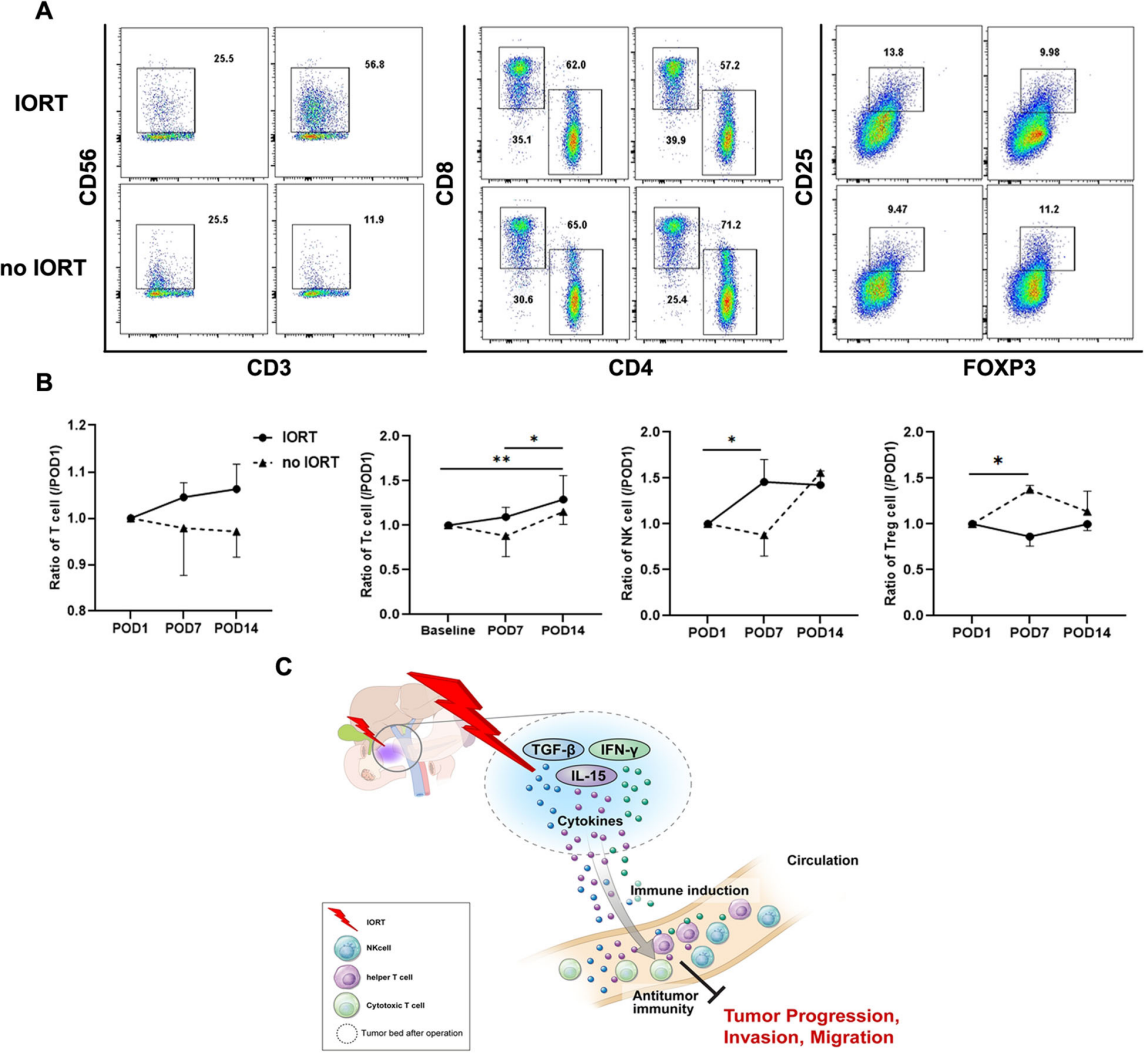
Changes in immune cell composition of PBMCs during the follow-up period of IORT-treated patients with pancreatic cancer. **A**. Representative flow cytometry plots illustrating the gating strategy used in the analysis of lymphocytes in the blood (left: CD3-CD56+ NK cell; middle: CD3 + CD4+ helper T cell and CD3 + CD8+ cytotoxic T cell; right: CD3-CD25 + FOXP3+ Treg cell). **B**. Rate of change in the indicated immune cells according to the blood collection time (baseline = post-operation day (POD) 1, normalised to each POD 1 value). **C**. Scheme of immune response by IORT treatment after pancreatic cancer surgery

Blood samples were collected from each patient group on POD 1, 7, and 14, after which flow cytometry was performed on the isolated PBMCs (Fig. 5A). The indicated immune cell population values on POD 7 and 14 were normalised to those on POD 1 (Fig. 5B). The total T cell population displayed a higher rate of increase during the 14-day post-operative period in the IORT PF group than in the no IORT group. Specifically, cytotoxic and helper T cell populations demonstrated significantly higher increasing rates in the IORT PF group than in the no IORT group. (Tc cell *p* = 0.0076, Th cell *p* = 0.0456) In the case of the NK cell population, a significantly higher increasing rate was observed during the first 7 days post-surgery in the IORT PF group than in the no IORT group. (*p* = 0.0216) Moreover, Treg cells maintained a reduced ratio throughout the 14-day post-operative period in the IORT PF group compared with that in the no IORT group. (*p* = 0.0242) These results indicated that IORT exerts a systemic effect on the microenvironment around the surgical site through the immune response.

## Discussion

Cytokines and growth factors secreted by tumour cells and surrounding cells are affected by radiation therapy and by invasive procedures, such as surgery [21, 22]. IFN, TGF-β, IL-1β, IL-6, IL-7, and granulocyte-macrophage colony-stimulating factor (GMCF) are known to regulate pro- and anti-immune responses [23]. In the current study, we identified cytokines secreted at elevated levels in the PF of IORT-treated patients with pancreatic cancer compared with those in the PF of patients who underwent surgical resection alone. Specifically, IFN-γ, TGF-β, IL-15, and PDGF-BB displayed significant differences between groups. TGF-β is a cytokine with dual effects on cancer because it exerts inhibitory cancer activity in the early stages of carcinogenesis but promotes cancer growth in the later stages [24, 25]. In particular, increased TGF-β secretion induces radio-resistance and is known to be a major modulator of the anti-cancer immune response during radiation therapy [26]. In our study, the TGF-β level was significantly increased in the IORT PF group, likely owing to radiation response [27]. Among the relatively highly expressed cytokines, IFN-γ is involved in mediating the anti-tumour effects of radiation therapy [28]. It is produced by related CD8+ T and NK cells through immune stimulation or inflammatory reactions, and subsequently affects the activity of these cells after radiation exposure [29]. Concurring with the results of previous studies, our data confirmed that post-operative IORT maintained a relatively high proportion of CD8+ T and NK cells in the blood and increased the IFN-γ level, which would lead to anti-cancer immune responses.

PDGF-BB, another relatively highly expressed cytokine, improves cell viability and induces cell proliferation and migration of several types of tumours. In accordance with our study results, PDGF-BB expression reportedly increases with TGF-β expression throughout the radiation process [24]. PI3K-Akt signalling induced by PDGF-BB is known to contribute to the migratory regulation of cancer progression [25]. Similar to the results of previous studies, PDGF-BB expression in the IORT PF group was elevated, and we confirmed that it affected PI3K-Akt signalling during PF treatment of pancreatic cancer cells. IL-15 is generally known to activate the innate immune system by inducing the differentiation and proliferation of NK cells [30]. In the present study, we confirmed its effect on PI3K-Akt signalling when pancreatic cancer cells were treated with PF, which concurred with similar results reported in a previous study [31]. Nevertheless, to confirm the effect of IORT on immune cells, further comparative response studies are needed. According to a previous study investigating IORT in breast cancer, levels of PF, IL-4, and IL-5, which are known to be increased by radiation, were elevated, whereas those of IL-6, RANTES, and HGF, which are known to control tumour cell growth and motility, were decreased in the surgical wound fluid of breast cancer patients [32]. In another breast cancer study, levels of IL-7, IL-8, IL-13, macrophage migration inhibitory factor (MIF), and TNF-β were increased, whereas those of CTACK, G-CSF, HGF, IL-1β, and TNF-α were decreased after IORT exposure [19]. Although our data did not demonstrate significant differences in cytokine levels, except for IL-8 and TNF-α, a similar trend in the data suggests that immunological effects are induced by IORT in pancreatic cancer. The differences displayed by cytokines in our study compared with those in previous studies highlight that pancreatic cancer has higher heterogeneity than other types of cancer, which may lead to different tendencies that could be reflected in the pancreatic cancer tumour [33].

The cytokines and chemokines present in PF affect the EMT process of cancer cells, and ultimately, invasion and metastasis. TGF-β1 reportedly induces the Smad4-dependent EMT transition in Panc1 cells, increasing the E-cadherin level while decreasing the N-cadherin and vimentin levels [34]. CXCR4 also activates the Wnt and Hedgehog signalling pathways to express the EMT phenotype and promotes CXCL12-mediated pancreatic cancer cell invasion and metastasis [35]. Belletti et al. first reported the stimulating role of post-operative fluid on the proliferation and migration of breast cancer cells [32]. In particular, Kulcenty et al. observed expression of the EMT phenotype and induction of cell migration when breast cancer cells were treated with post-operative fluid, which was abrogated by IORT treatment [36]. In the present study, proliferation, invasion, and motility of all three pancreatic cell lines were stimulated by PF, the effects of which were suppressed in cells stimulated with IORT-treated PF. Epithelial and mesenchymal markers in pancreatic cancer cells were used to confirm inhibition of the EMT process, revealing the same pattern as the functional assay. These results are consistent with the above-mentioned studies, indicating that IORT-induced changes in the tumour microenvironment are responsible for suppression of the proliferation, invasiveness, and migratory ability of pancreatic cancer cells.

Analysing blood samples after surgery, we confirmed that the ratio of anti-tumour immune cells on POD 1 was decreased in most patients compared with that before surgery but was gradually recovered to pre-operative levels over time (data not shown). Hence, we compared the proportion of immune cells that changed over time based on the levels measured on POD 1. Cytotoxic T cell and NK cell populations demonstrated a tendency towards higher increasing rates after surgery among patients who received IORT. Conversely, the abundance of Treg cells, which are immune suppressive cells, remained low in patients who received IORT. Irradiation of the cavity from which the tumour is removed causes apoptosis in the few remaining tumour cells and lymphocytes, and secretion of DAMP, tumour antigens, and cytokines, consequently establishing an environment that attracts immune cells, called the “bystander effect” [37, 38]. Our results support the formation of an inflammatory microenvironment induced by high-dose irradiation that can trigger an anti-tumour immune response in pancreatic cancer through the bystander effect. Pancreatic cancer is a representative “cold tumour”; the implementation of IORT may contribute to a positive feedback loop that continuously activates immune-related cells, thereby forming an immune environment that would improve local control of pancreatic cancer.

Drainage insertion after pancreatic cancer surgery is controversial; nevertheless, many surgeons perform draining because of complications caused by pancreatic fistulas [39]. Drainage fluid collected from the abdominal cavity of patients with pancreatic cancer has been used as a diagnostic indicator of pancreatic leaks or ascites, unlike wound fluid from breast cancer surgery [40, 41]. Various cancers, including pancreatic cancer, are known to protect malignant cells and accumulate fluid (malignant effusion) through the secretion of cytokines, growth factors, or peptides [42]. Several studies have investigated the tumour microenvironment through PF analysis for other cancer types, but little research has been conducted on PF in pancreatic cancer to date [43, 44]. The present study improves our understanding of the pancreatic cancer tumour microenvironment through component analysis and related signalling pathways in the PF, with respect to IORT after pancreatic cancer surgery. However, further evaluation is needed to compare serum cytokine levels and more accurately correlate immune responses altered by IORT.

The local recurrence rate of pancreatic cancer is high even after radical surgery and adjuvant chemotherapy [2]. In our study, among patients who underwent surgery, local recurrence occurred in 31.3% (5/16) of patients who received IORT and 50% (5/10) of those who did not receive IORT (Supplementary Table S4). In addition, when the 1-year disease-free survival rate was analysed based on the 2-year follow-up data, it was confirmed that the IORT group had a higher rate (Supplementary Fig. S1). Although they did not reach statistical significance, these findings support ​​disease-free survival and local recurrence benefits via IORT of pancreatic cancer.

This study has several limitations that should be acknowledged when interpreting the results. First, this was a prospective study with nonrandomised patients because it was classified according to patient consent. Second, because the PF used in this study was derived from the entire abdominal cavity, it is possible that the other factors such as stress and inflammation from surgery, in addition to IORT, may have influenced the results. Third, only the indirect immune effects by IORT could be confirmed through the ratio analysis of PBMC cells using blood.

## Conclusion

This is the first study to employ blood and PF to confirm the effect of IORT on pancreatic cancer cells and the anti-cancer immune response after surgical resection. We identified differentially expressed cytokines in PF and compared the effect of IORT administration on the proliferation and activity of pancreatic cancer cells stimulated by PF. We also confirmed the anti-cancer immune response induced by IORT through comparison of immune cell populations during the post-operative period. Through this study, we can conclude that various cytokines at the surgical site induce microenvironment changes after IORT, which inhibit the proliferation of remaining cancer cells and recurrence (Fig. 5C). Therefore, IORT induces an anti-cancer immune response in patients with pancreatic cancer, ultimately aiding local control and prevention of pancreatic cancer recurrence.

## Supplementary Information

**Additional file 1 Supplementary Table S1.** Summary of patients’ clinical characteristics.

**Additional file 2 Supplementary Table S2.** Clinical characteristic of 30 patients.

**Additional file 3 Supplementary Table S3.** Cytokine levels in PF altered by IORT.

**Additional file 4 Supplementary Table S4.** Recurrence pattern of patients with pancreatic cancer.

**Additional file 5 Supplementary Fig. S1.** 1-year disease free survival rate according to IORT.

## Data Availability

The datasets used and/or analysed during the current study are available from the corresponding author on reasonable request.
