# Supplementary figures and images for: Intraoperative radiation therapy induces immune response activity after pancreatic surgery
Source: BMC Cancer. 2021 Oct 12;21:1097. doi: 10.1186/s12885-021-08807-3 (PMC8507125; doi:10.1186/s12885-021-08807-3)

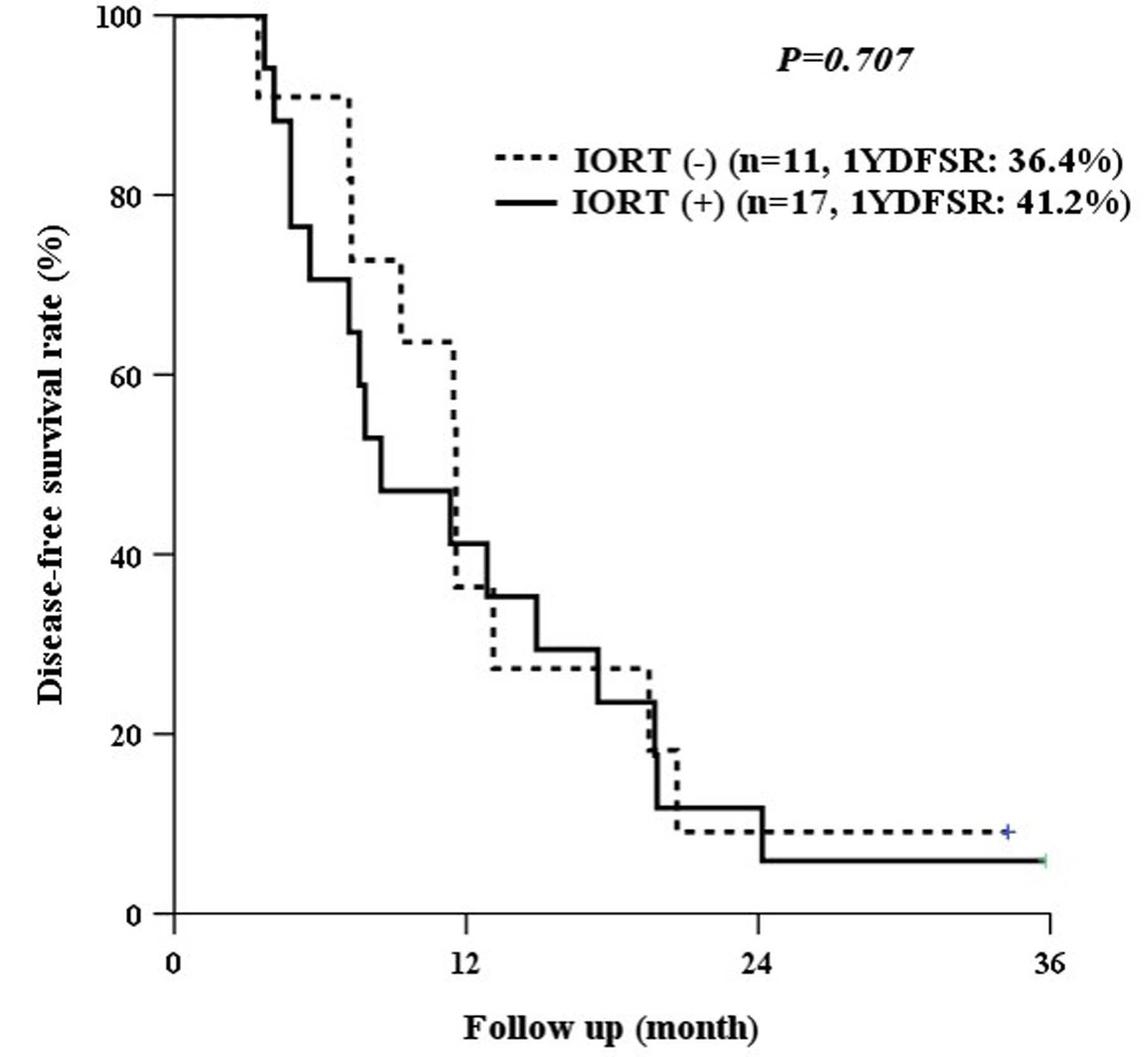

Supplement: Supplementary file 5 — Additional file 5 Supplementary Fig. S1. 1-year disease free survival rate according to IORT. [file 12885_2021_8807_MOESM5_ESM.jpg]
